# Direct Current Cardioversion in Atrial Fibrillation Patients on Edoxaban Therapy Versus Vitamin K Antagonists: a Real-world Propensity Score–Matched Study

**DOI:** 10.1007/s10557-020-07078-7

**Published:** 2020-09-18

**Authors:** Anna Rago, Andrea Antonio Papa, Emilio Attena, Valentina Parisi, Paolo Golino, Gerardo Nigro, Vincenzo Russo

**Affiliations:** 1 University of the Study of Campania “Luigi Vanvitelli” – Monaldi Hospital, P.zzale E. Ruggeri, 80131 Naples, Italy; 2 Department of Cardiology, Health Authority Naples, Naples, Italy; 3 Department of Translational Medical Sciences, University of Naples Federico II, Naples, Italy

**Keywords:** Atrial fibrillation, Transesophageal echocardiogram, Direct electrical current cardioversion, Edoxaban, Uninterrupted vitamin K antagonists

## Abstract

**Purpose:**

The purpose of the present study was to compare the long-term effectiveness and safety of newly initiated anticoagulation with edoxaban (EDO) versus uninterrupted vitamin K antagonist (VKA) therapy in patients with atrial fibrillation (AF) scheduled for transesophageal echocardiogram (TEE)-guided direct electrical current cardioversion (DCC).

**Methods:**

A propensity score-matched cohort observational study was performed comparing the safety and effectiveness of edoxaban versus well-controlled VKA therapy among a cohort of consecutive non-valvular AF patients scheduled for DCC. The primary safety outcome was major bleeding. The primary efficacy outcome was the composite of stroke, transient ischemic attack (TIA), and systemic embolism (SE).

**Findings:**

A total of 130 AF patients receiving edoxaban 60-mg (EDO) treatment were compared with the same number of VKA recipients. The cumulative incidence of major bleedings was 1.54% in the EDO group and 3.08% in the VKA group (*P =* 0.4)*.* The cumulative incidence of thromboembolic events was 1.54% in the EDO group and 2.31% in the VKA group (*P =* 0.9)*.* A non-significant trend in improved adherence was observed between the EDO and VKA groups with a total anticoagulant therapy discontinuation rate of 4.62% (6/130) vs 6.15% (8/130), respectively (*P =* 0.06).

**Implications:**

Our study provides the evidence of a safe and effective use of edoxaban in this clinical setting, justified by no significant difference in major bleedings and thromboembolic events between edoxaban and well-controlled VKA treatments.

## Introduction

Cardioversion (both electric and pharmacological) in AF patients is associated with an increased risk of thromboembolic events [1, 2] and an adequate level of periprocedural anticoagulation is essential to reduce this risk. The use of direct oral anticoagulants (DOACs) in clinical practice is rapidly increasing [3–9], even in the setting of patients with AF undergoing electrical cardioversion [10–13], and the current guidelines recommend initiating anticoagulation with DOACs as soon as possible before every cardioversion of AF [14, 15]. For patients with AF of > 48-h duration, the current recommendation is to start oral anticoagulation at least 3 weeks before cardioversion and continue it for at least 4 weeks afterwards. Edoxaban (EDO), a direct oral factor Xa inhibitor, was demonstrated to be non-inferior to enoxaparin-warfarin therapy in terms of composite net clinical benefit outcome of stroke, systemic embolic event, transient ischemic attack, myocardial infarction, cardiovascular mortality, and major bleeding events in patients undergoing cardioversion of atrial fibrillation [16]. However, there are no real-world data on clinical outcomes following direct current cardioversion (DCC) in AF patients treated with edoxaban. The aim of the present study was to investigate the safety and effectiveness of newly initiated anticoagulation with edoxaban versus uninterrupted vitamin K antagonists (VKA) therapy in patients with non-valvular AF scheduled for transesophageal echocardiography (TEE)-guided DCC.

## Materials and Methods

### Database

Atrial Fibrillation Research Database (NCT03760874) is a prospectively maintained database shared by three cardiologic centers in Italy (Monaldi Hospital, Naples; University of Campania “Luigi Vanvitelli”, Naples; Departement of Cardiology, Health Authority Naples) which includes all patients with non-valvular AF followed at our institution between January 2017 and January 2019. At each 6-month follow-up visit, the clinical status, occurrence of stroke, transient ischemic attack (TIA), systemic embolism (SE), myocardial infarction, major bleeding (MB), clinically relevant non-major bleedings (CRNMB), or other side effects were assessed. Informed consent was obtained from all participants before inclusion in the database. The database and the present analysis were approved by the local institutional review committee.

### Definitions

Ischemic stroke was defined as an episode of neurologic deficit lasting > 24 h in the absence of an intracranial hemorrhage and diagnosed by cerebral computerized tomography. TIA was defined as a temporary neurologic deficit lasting < 24 h. SE was defined as an acute vascular occlusion of an extremity or organ. MB was defined as that which was clinically overt and associated with any of the following: fatal outcome, involvement of a critical anatomic site, fall in hemoglobin ≥ 2 g/dL, transfusion of > 2 U of whole blood, or packed red blood cells, according to the International Society on Thrombosis and Hemostasis (ISTH) criteria [17]. CRNMB was defined as overt bleeding not meeting the criteria for MB but requiring medical intervention, unscheduled contact (visit or telephone) with a physician, temporary interruption of study drug (i.e., delayed dosing), pain, or impairment of daily activities according to ISTH criteria [17]. Well-controlled VKA therapy was defined as VKA treatment with high time in therapeutic range (> 70%).

### Patient Population

The database was queried for patients with persistent AF who underwent TEE-guided DCC during the time period from January 2017 to January 2019 who received edoxaban or VKA treatment. Patients with a follow-up ≤ 360 days from the first qualifying anticoagulant prescription and VKA with time in therapeutic range < 70% have been excluded from the analysis, in order to perform an accurate analysis of long-term safety and effectiveness of optimal anticoagulant therapy in real-world AF patients. Propensity score matching analysis generated two groups (edoxaban vs VKA) with minimal differences in baseline characteristics.

### Outcomes

The occurrence of MB events, including the intracranial hemorrhage (ICH), was the primary safety outcome. Occurrence of TE was the primary effectiveness outcome. The secondary effectiveness outcome included death from any cause; the secondary safety outcome included CRNMB events.

### Statistical Analysis

The Kolmogorov-Smirnov normality test was used to analyze data normality. Continuous variables were reported using the mean and standard deviation. Categorical variables were indicated as frequency counts and percentages. Baseline characteristics between edoxaban and VKA groups were compared by *t* test for continuous variables and Chi-squared test for categorical variables. Nearest neighbor propensity score matching with 1:1 ratio was used to minimize bias between VKA and edoxaban groups. The variables included in the propensity score were age, female sex, BMI, hypertension, CHA_2_DS_2_-VASc score, HAS-BLED score, diabetes mellitus, heart failure, prior stroke/TIA, prior myocardial infarction (MI), glomerular filtration rate, left atrial diameter, indexed left atrial volume, and left ventricle ejection fraction. The incidence of thromboembolic and bleeding events was calculated both as incidence rate every 100 patient-years and as cumulative incidence. We considered as statistically significant all *P* values less than 5%. All statistical analyses were performed using Rstudio (RStudio Team (2016). RStudio: Integrated Development for R. RStudio, Inc., Boston, MA URL http://www.rstudio.com/).

## Results

We identified 495 consecutive patients with persistent AF scheduled for TEE-guided DCC during the time period from January 2017 to January 2019 who received edoxaban (*n* = 230) or VKA treatment (*n* = 210). We excluded patients with a follow-up ≤ 360 days from the first qualifying anticoagulant prescription (*n* = 20) and VKA patients with time in therapeutic range < 70% (*n* = 35).

Propensity score matching identified 130 edoxaban and the same number of VKA recipients scheduled for DCC who were comparable with respect to demographic and clinical characteristics. Baseline characteristics of the study population before and after propensity score matching are summarized in Table 1. The mean follow-up was 20 ± 4 months. Among overall VKA patients before propensity score matching (*n* = 210), the INR tests were performed every 10 days; therefore, about 12,600 INR tests were overall performed during the observational period.

**Table 1:** Baseline characteristics of the study population before and after propensity matching

Variable	Before propensity score matching			After propensity score matching		
	EDO (*n* = 230)	VKA (*n* = 210)	*P* value	EDO (*n* = 130)	VKA (*n* = 130)	*P* value
Age (years)	61.1 ± 10.4	73.9 ± 6.4	< 0.001	62.3 ± 10.2	63.1 ± 10.3	0.73
Female (%)	46.4	42.1	0.58	42.5	41.8	0.71
BMI (kg/m^2^),	27.8 ± 4.9	29.8 ± 6.1	0.82	26.9 ± 7.3	26.8 ± 7.2	0.77
Hypertension (%)	49.5	59.2	0.001	54.2	56.1	0.57
CHA2DS2VASc score	2.5 ± 1.7	3.3 ± 1.3	0.02	2.1 ± 0.6	2.2 ± 0.4	0.55
HAS-BLED score	2.2 ± 1.1	3.2 ± 1.0	0.001	2.05 ± 1.1	2.04 ± 1.5	0.55
Diabetes mellitus (%)	15	22	0.06	14	13	0.4
Heart failure (%)	17.7	27.1	0.001	21.6	22.3	0.8
Prior stroke/TIA (%)	26.9	36.4	0.001	27.8	28.3	0.6
Prior MI (%)	8.2	13.1	0.02	5.6	6.2	0.7
CrCl (mL/min)	72.3 ± 14.1	60.7 ± 12.9	0.001	71.3 ± 21.1	72.2 ± 21.2	0.7
Left atrial diameter (mm)	44.3 ± 6.7	46.8 ± 5.7	0.8	46.2 ± 5.3	47.1 ± 5.7	0.8
LAVI (mL/m^2^)	32.3 ± 1.1	33.7 ± 1.5	0.42	32.3 ± 2.4	33.2 ± 1.2	0.7
LV EF (%)	53.4 ± 6.5	44.3 ± 6.1	0.002	55.4 ± 5.2	54.3 ± 3.9	0.8
TEE performed (%)	100	100		100	100	

Values are expressed as mean ± SD unless otherwise stated

*BMI* body mass index, *CrCl* creatinine clearance, *EDO* edoxaban, *LAVI* indexed left atrial volume, *MI* myocardial infarction, *SD* standard deviation, *TIA* transient ischemic attack, *VKA* vitamin K antagonist, *LV EF* left ventricle ejection fraction, *TEE* transesophageal echocardiogram

The TEE revealed the presence of a left atrial appendage thrombus in two patients. These patients showed high thromboembolic risk and moderate renal impairment: one patient (0.7%) in the edoxaban group (30 mg OD; CHA2DS2VASc score: 5; and glomerular filtration rate (GFR): 38 mL/min) and one patient (0.7%) in the VKA group (INR: 2.1; CHA2DS2VASc score: 4 and GFR: 35 mL/min). The DCC was performed within the target time range of 23 ± 2 days. The acute cardioversion success rate was 86.9% (*n* = 113/130) in the edoxaban group and 83.8% (*n* = 109/130) in the VKA group (*P =* 0.4)*.* Four weeks after DCC, all patients continued taking anticoagulant therapy due to having a CHA2DS2VASc score ≥ 2.

A total of six patients among study population (two in EDO and four in VKA) experienced primary safety outcome. The cumulative incidence of major bleedings was 1.54% in the EDO group and 3.08% in the VKA group (*P =* 0.4)*.* A total of five patients among study population (two in EDO and three in VKA) experienced primary effectiveness outcome. The cumulative incidence of thromboembolic events was 1.54% in the EDO group and 2.31% in the VKA group (*P =* 0.9)*.* A total of three patients died among study population (one in EDO and two in VKA). The cumulative incidence of all-cause mortality was 0.77% in the EDO group and 1.54% in the VKA group (*P =* 0.06)*.* A cardiovascular death occurred in a patient receiving VKA; two non-cardiovascular deaths were reported (one lung cancer-related death in the EDO group and one bladder cancer-related death in the VKA group). A total of 14 patients (5 in EDO and 8 in VKA) experienced secondary safety outcome. The cumulative incidence of CRNMB events was 3.8% in the EDO group and 6.1% in the VKA group (*P =* 0.03)*.* The total anticoagulant therapy discontinuation rate was 4.62% (6/130) in the EDO group and 6.15% (8/130) in the VKA group (*P =* 0.06) (Table 2).

**Table 2:** Efficacy and safety endpoints and therapy discontinuation rate in the EDO and VKA groups

Variable			
	EDO	VKA	*P* value
	(*n* = 130)	(*n* = 130)	
Stroke/TIA/SE	1.54%	2.31%	0.9
Major bleedings	1.54%	3.08%	0.4
All-cause death	0.77%	1.54%	0.06
CRNMB	3.8%	6.1%	0.03
Therapy discontinuation rate	4.62%	6.15%	0.06

*TIA* transient ischemic attack, *SE* systemic embolism, *CRNMB* clinically non-relevant non-major bleedings

## Discussion

Real-world evidences on clinical performance of non-vitamin K antagonist oral anticoagulant (NOACs) use in AF patients scheduled for DCC are relevant to address current unmet medical needs and to better define the specific role of such agents in this clinical setting. NOACs offer several potential advantages over VKAs in AF patients undergoing cardioversion, removing the need for routine laboratory monitoring and heparin bridging therapy and reducing time to procedure [18]. The present study is the first real-world experience investigating the safety and effectiveness of newly initiated anticoagulation with edoxaban versus well-controlled VKA therapy in patients with non-valvular AF scheduled for TEE-guided DCC. Our results did not show a statistically significant difference in the cumulative incidence of both major bleedings and thromboembolic events among AF patients who underwent DCC on edoxaban vs VKA. Our data confirm the results of the ENSURE AF study [16], which showed in 2199 AF patients undergoing DCC a similar cumulative incidence for the composite net clinical benefit outcome of stroke, systemic embolic event, transient ischemic attack, myocardial infarction, cardiovascular mortality, and major bleeding events between edoxaban group vs enoxaparin-VKA group. Moreover, our study showed a low incidence of left atrial thrombus revealed by TEE in AF patients on edoxaban therapy (0.7%), similar to that observed in the VKA study group (0.7%), but lower than those reported by ENSURE-AF prospective randomized study [16], in which the authors identified left atrial appendage thrombus by TEE in 47 patients (8%) in the edoxaban group and in 42 patients (7.1%) in the enoxaparin–warfarin group. The low left atrial thrombus incidence reported by the present study was similar to those reported by previous real-world studies analyzing dabigatran (0.6%) and apixaban (0.5%) clinical performances in AF patients undergoing DCC [11, 13]. These results may be explained by the different clinical characteristics of our study population compared with those of previous randomized clinical trials (RCTs); our study population showed that mean CHA_2_DS_2_-VASc score values were lower (2.1 ± 0.6 in EDO group vs 2.2 ± 0.4 in VKA group) than those (([2–6 (SD 1·4)) reported by Ensure [16], and similar to values reported by other previous real-world experiences [11, 13]. The presence of left atrial appendage thrombus at TEE in two patients with thromboembolic risk (CHA_2_DS_2_-VASc score ≥ 4) and moderate renal impairment (GFR < 40 mL/min) supports the hypothesis that these factors might be considered predictors of left atrial thrombus in the real-world setting [11, 13, 19, 20].

### Limitations

The present is a single-centre observational study mainly including low-risk patients with no history of stroke or major bleeding; this is because we usually propose in our clinical practice the rhythm control strategy at the first episode of persistent AF. Our results should be verified in AF patients at moderate-high risk of stroke/major bleeding. Moreover, we compared two groups with several different clinical characteristics; however, the propensity score matching has been used to account the differences. Having a sample size determined by propensity score matching and comparing two proportions with an equivalent test, we decided to set a type I error at 5% and a non-inferiority margin at 4%. Therefore, in the case of cumulative incidence of major bleedings (1.54% in EDO group, 3.08% in VKA group), we have a power of 62%; in the case of cumulative incidence of thromboembolic events (1.54% in EDO group and 2.31% in VKA group), we have a power of 69%. The observation and reporting of adverse effects are usually more accurate and careful in randomized controlled trials than in clinical practice; thus, further studies with wider sizes would be required to detect any differences, if present, in thromboembolic and hemorrhagic events between patients receiving edoxaban and undergoing electrical cardioversion for AF and those on continuous VKA, given the low incidence of thromboembolic and hemorrhagic events with either strategy.

## Conclusion

There are no real-world data on safety and effectiveness of edoxaban in patients undergoing TEE-guided direct current cardioversion. Our study provides the evidence of a safe and effective use of edoxaban in this clinical setting, justified by no significant difference in major bleedings and thromboembolic events between edoxaban and well-controlled VKA treatments.
